# CXCR4 Expression in Prostate Cancer Progenitor Cells

**DOI:** 10.1371/journal.pone.0031226

**Published:** 2012-02-16

**Authors:** Anna Dubrovska, Jimmy Elliott, Richard J. Salamone, Gennady D. Telegeev, Alexander E. Stakhovsky, Ihor B. Schepotin, Feng Yan, Yan Wang, Laure C. Bouchez, Sumith A. Kularatne, James Watson, Christopher Trussell, Venkateshwar A. Reddy, Charles Y. Cho, Peter G. Schultz

**Affiliations:** 1 The Scripps Research Institute, La Jolla, California, United States of America; 2 Genomics Institute of the Novartis Research Foundation, San Diego, California, United States of America; 3 National Cancer Institute, Kyiv, Ukraine; 4 Institute of Molecular Biology and Genetics NAS of Ukraine, Kyiv, Ukraine; Children's Hospital & Harvard Medical School, United States of America

## Abstract

Tumor progenitor cells represent a population of drug-resistant cells that can survive conventional chemotherapy and lead to tumor relapse. However, little is known of the role of tumor progenitors in prostate cancer metastasis. The studies reported herein show that the CXCR4/CXCL12 axis, a key regulator of tumor dissemination, plays a role in the maintenance of prostate cancer stem-like cells. The CXCL4/CXCR12 pathway is activated in the CD44^+^/CD133^+^ prostate progenitor population and affects differentiation potential, cell adhesion, clonal growth and tumorigenicity. Furthermore, prostate tumor xenograft studies in mice showed that a combination of the CXCR4 receptor antagonist AMD3100, which targets prostate cancer stem-like cells, and the conventional chemotherapeutic drug Taxotere, which targets the bulk tumor, is significantly more effective in eradicating tumors as compared to monotherapy.

## Introduction

Prostate cancers are the second most common cause of cancer death in men. Most prostate cancers are hormone dependent and respond to androgen ablation therapy. However, ablation therapy is not curative for metastatic prostate cancers, because these tumors eventually become hormone refractory and grow despite androgen ablation, even though initial treatment appeared successful [1], [2]. Chemotherapy regimens that include the drug docetaxel (Taxotere) extend median survival by two to three months in patients with advanced prostate cancer that is no longer responsive to hormone therapy. Nevertheless, with a 5 year survival rate of 17% patients and a median survival period of 2–3 years, the prognosis for patients with metastatic prostate cancer remains poor [3].

It has been argued that tumor progenitor cells play a crucial role in tumor development and represent a drug resistant cell population that can survive conventional treatment and cause disease relapse [4]. This notion suggests that therapies targeting tumor progenitors may lead to more effective cancer treatments [5], [6]. Cell populations expressing the surface markers CD133 and CD44 have been identified as putative stem cell populations in the prostate gland [5], [7], [8], [9]. We and others have demonstrated that CD133^+^/CD44^+^ cells from established prostate cancer cell lines are also self-renewing and multipotent, and have strong tumorigenic potential *in vivo* [5], [7], [10], [11], [12]. Whereas prostate cancer cell lines cultured under long-term monolayer culture conditions contain less than 2% prostate cancer progenitors, this CD133^+^/CD44^+^ cancer progenitor population can be expanded under anchorage independent serum-free conditions (sphere forming conditions) [5], [11]. This enriched progenitor population maintains increased self-renewal capacity and tumorigenicity [5]. mRNA expression analysis revealed that the chemokine receptor CXCR4 is highly upregulated in these cells compared to cells cultured under monolayer growth conditions [5]. It is known that activation of the CXCR4/CXCL12 pathway alters the adherence, migration, and invasion of cancer cells, including prostate cancer [13], [14], [15]. However, little is known about the role of this pathway in the maintenance of prostate tumor initiating cell populations. Herein we report studies that suggest the CXCR4/CXCL12 axis is activated in prostate cancer progenitors and plays a role in self-renewal, differentiation potential, cell adhesion, and tumorigenicity. Moreover, mouse xenograft studies suggest that inhibition of the CXCR4 pathway may be beneficial in the targeting of prostate cancer progenitors *in vivo*.

## Results

### The CXCL4/CXCR12pathway is activated in the CD44^+^/CD133^+^prostate progenitor population

**Figure 1:**
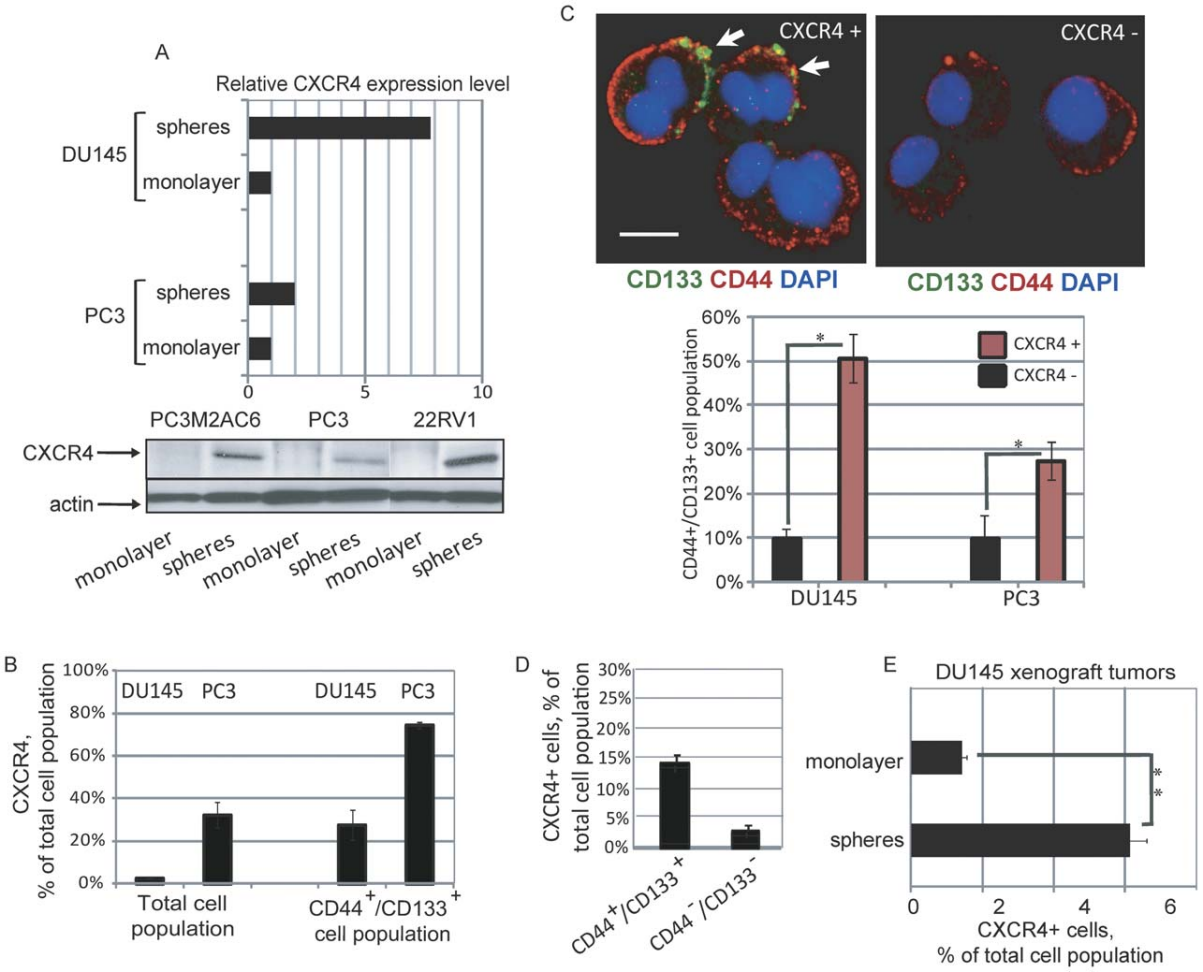
Overexpression of CXCR4 inprostate cancer progenitors. (A) Cells grown under sphere forming conditions showed an increased expression level of CXCR4 as analyzed by RT-PCR and Western blot analysis. For sphere formation, single cells were plated at 500 cells/mL in 10-cm dishes with an ultralow attachment surface and grown in serum-free epithelial basal medium for 7 days. (B) Flow cytometry analysis showed significant enrichment of the CXCR4^+^ population within CD44^+^/CD133^+^ cells compared to the total cell population for DU145 and PC3 cells (p<0.001). The cells were triple stained and analyzed with a BD LSR II flow cytometer. (C) Representative fluorescent images of CD133 and CD44 co-immunostaining showing that CXCR4^+^ DU145 and PC3 cells have a higher proportion of CD44^+^/CD133^+^ cells compared to CXCR4^−^ cells. Cells expressing high of low levels of CXCR4 were FACS-purified, plated in 384 well black clear bottom plates at a density of 100 cells/well in serum-free epithelial basal medium. After 18 hours, the cells were fixed with 3.7**%** formaldehyde in PBS and stained with anti-CD133 and anti-CD44 antibodies. Cells in at least five randomly selected fields of view were counted for each condition. Arrows show the triple positive cells. Scale bars indicate 15 µm. *- p value<0.05. (D) Immunostaining of paraffin-embedded sections of xenograft tumors formed by FACS purified CD44^+^/CD133^+^ and CD44^−^/CD133^−^ cells showed more than 13% CXCR4^+^ cells in tumors derived from CD44^+^/CD133^+^ cells compared to 2.2% CXCR4^+^ cells in xenograft tumors derived from CD44^−^/CD133^−^ cells. A total of 10^3^ FACS-sorted DU145 CD133^+^/CD44^+^ or CD133^−^/CD44^−^ cells embedded in BD matrigel were injected s.c. into NOD/SCID mice. Tumors were allowed to grow for 42 days until the tumors produced by DU145 CD133^+^/CD44^+^ reached a size of 400 mm^3^ and the tumors produced by DU145 CD133^−^/CD44^−^ reached a size of 125 mm^3^. Cells in at least five randomly selected fields of view were counted for each condition. Scale bars indicate 30 µm. (E) CXCR4 immunostaining on paraffin-embedded sections of xenograft tumors made by the cells grown under sphere forming and monolayer conditions showed more than 6% CXCR4^+^ cells in sphere-derived tumors as compared to 1.4% of CXCR4^+^ cells in monolayer-derived xenograft tumors. **- p value<0.01.Scale bars indicate 30 µm.

Several putative stem cell populations have been identified in prostate and are characterized by the cell surface markers CD44, CD133, and integrin α2β1^high^ [5], [7], [8], [9], [10], [11], [12]. Prostate cancer cells expressing CD44 and CD133 cell surface markers can be enriched from prostate cancer cell lines by growing them as spheres in progenitor media conditions [5]. We and others have confirmed that this CD133^+^/CD44^+^ cell population makes up a subset of prostate cancer cells that are self renewing, differentiate into heterogeneous tumors, and are highly tumorigenic in immunodeficient mice [5], [7], [8], [9], [10], [11], [12]. To identify signaling pathways selectively activated in this CD133^+^/CD44^+^ population, we carried out microarray analysis of gene expression in DU145 and PC3 cells using Affymetrix U133 arrays [5]. Gene expression profiling showed that the chemokine receptor CXCR4 is highly upregulated in both cell lines grown under sphere forming compared to monolayer growth conditions (a 13-fold increase and 104.6-fold increase for PC3 and DU145 cells, respectively) [5]. The high level of CXCR4 expression observed in microarray experiments was confirmed by quantitative RT-PCR and Western blot analysis (Figure 1A).

A growing body of evidence has demonstrated that CXCR4 plays an important role in cancer proliferation, dissemination, invasion and drug resistance [13], [14], [15], [16], [17], [18], [19], [20], [21], [22]. However, little is known about the role of the CXCR4/CXCL12 signaling pathway in the maintenance of prostate cancer progenitors. To verify that CXCR4 expression is upregulated in prostate tumor initiating cells, we examined CXCR4 levels in CD44^+^/CD133^+^ prostate cancer progenitor cells and total cell populations in the DU145 and PC3 cell lines by flow cytometry. We observed a 10.3- and 2.3-fold enrichment in the percentage of CXCR4^+^ cells in the CD44^+^/CD133^+^ population compared to the percentage of CXCR4^+^ cells in the total population of DU145 and PC3 cells, respectively (Figure 1B). Consistent with this observation, FACS-purified DU145 CXCR4^+^ and PC3 CXCR4^+^ populations have a considerably higher proportion of CD133^+^/CD44^+^ cells as compared to CXCR4^−^ cells (a 5.8-fold and 2.8-fold increase for DU145 and PC3 cells, respectively) (Figure 1C). This increased level of CXCR4 expression in the CD133^+^/CD44^+^ cell population suggests an important role for CXCR4 signaling in the maintenance of prostate cancer progenitors.

To determine whether the same correlation exists *in vivo* we analyzed CXCR4 expression in tumors from mice injected with CD133^+^/CD44^+^ enriched cells as well as cells grown under sphere forming conditions. Both prostate cell populations have previously been shown to have increased tumorigenicity [5], [12]. Histological analysis of DU145 xenograft tumors derived from FACS-purified CD44^+^/CD133^+^ cells revealed a significantly higher CXCR4^+^ cell population than in tumors formed by CD44^−^/CD133^−^ cells (Figure 1D). Similarly, analysis of CXCR4 expression in xenograft tumors from mice injected with DU145 cells grown under sphere or monolayer conditions showed that CXCR4 expressing cells make up 6.1% of the total cell population in sphere-derived tumors, whereas monolayer-derived tumors have 1.4% of CXCR4 positive cells (Figure 1E). Thus, a higher percentage of cells expressing CXCR4 is also associated with tumors derived from both CD44^+^/CD133^+^ cells or cells grown under sphere forming conditions.

**Figure 2:**
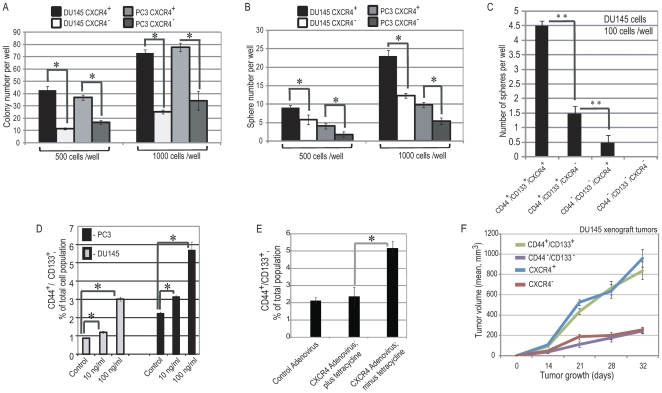
CXCR4/CXCL12 signaling pathway plays an important role in the maintenance of prostate cancer progenitor cells. CXCR4^+^ DU145 and PC3 cells showed an increase in (A) clonogenicity and (B) sphere forming ability over CXCR4^−^ cells. (C) CD44^+^/CD133^+^/CXCR4^+^ DU145 cells showed an increase in sphere forming ability compared to CD44^+^/CD133^+^/CXCR4^−^ cells. The cells were purified by FACS and plated in 24-well low attachment plate at a density of 100 cells/well in serum-free epithelial basal medium and grown for 10 days. **- p value<0.01. (D) Flow cytometry analysis showed that CXCL12 stimulates proliferation of prostate cancer progenitors in DU145 and PC3 cell lines in a dose-dependent manner. DU145 and PC3 cells were grown in serum-free, EBM medium with supplements and treated with the indicated concentrations of CXCL12 replenished daily for 5 days. (E) Overexpression of CXCR4 in prostate cancer cells resulted in a 3-fold increase of CD44^+^/CD133^+^ population. DU145 cells were infected with adenovirus encoding CXCR4 under the control of the tet-off regulatory system, and with control adenovirus and analyzed 4 days after infection. CXCR4 expression was validated by flow cytometry analysis. (F) CXCR4^+^ cells possess higher tumorigenic properties compared to CXCR4^−^ cells. 10^3^ CXCR4^+^, CXCR4^−^, CD44^+^/CD133^+^ and CD44^−^/CD133^−^ DU145 cells collected by FACS sorting were embedded in BD matrigel and injected s.c. into NOD/SCID mice. Each experimental group contained at least five mice. Error bars represent SEM.

Next, we analyzed the relationship between CXCR4 expression and the self renewal capacity and tumorigenicity of prostate cancer progenitor cells. Both FACS sorted DU145 and PC3 CXCR4^+^ populations showed an increase in sphere and colony forming potential over CXCR4^−^ cells (2.3-fold increase and 3.9-fold increase, respectively) (Figure 2A, B). Similarly, CD44^+^/CD133^+^/CXCR4^+^ cells have higher spherogenic potential as compared to CD44^+^/CD133^+^/CXCR4^−^ cells (Figure 2C). To evaluate the self-renewal capacity of CXCR4^+^ cells, secondary spheres were generated from dissociated primary spheres derived from PC3 CXCR4^+^ and PC3 CXCR4^−^ cells (Figure S1A). The number of secondary spheres per 1000 or 500 cells was higher with spheres derived from CXCR4^+^ cells than from CXCR4^−^ cells (a 5.5-fold increase and 3.2-fold increase, respectively). To determine whether activation of the CXCR4/CXCL12 axis stimulates proliferation of prostate cancer progenitors, PC3 and DU145 cells were treated with CXCL12 at 10 and 100 ng/mL for 5 days in serum-free epithelial growth medium. As shown in Figure 2D, activation of the CXCR4/CXCL12 signaling pathway significantly increases the CD44^+^/CD133^+^ population for both PC3 and DU145 prostate cancer cell lines in a dose dependent manner (up to 3.5-fold and 2.6-fold increase, respectively). Similarly, adenovirus-mediated overexpression of CXCR4 in DU145 cells resulted in a more than 2.5-fold increase of the CD44^+^/CD133^+^ population (Figure 2E and Figure S1B), but had little effect on the CD44^−^/CD133^−^ and total cell populations. To further characterize the tumor forming potential of CXCR4^+^ cells, 1000 FACS-purified CXCR4^+^ and CXCR4^−^DU145 cells were injected subcutaneously into NOD/SCID mice. CXCR4^+^ cells were found to have significantly higher tumorigenicity than CXCR4^−^ cells (up to 3.8-fold increase in xenograft tumor growth, Figure 2F, Figure S1C). In fact the CD44^+^/CD133^+^ and CXCR4^+^ cells exhibited a similar tumorigenic potential *in vivo*. These results indicate that expression of CXCR4 contributes to tumorigenic potential of androgen refractory prostate cancer cell lines.

**Figure 3:**
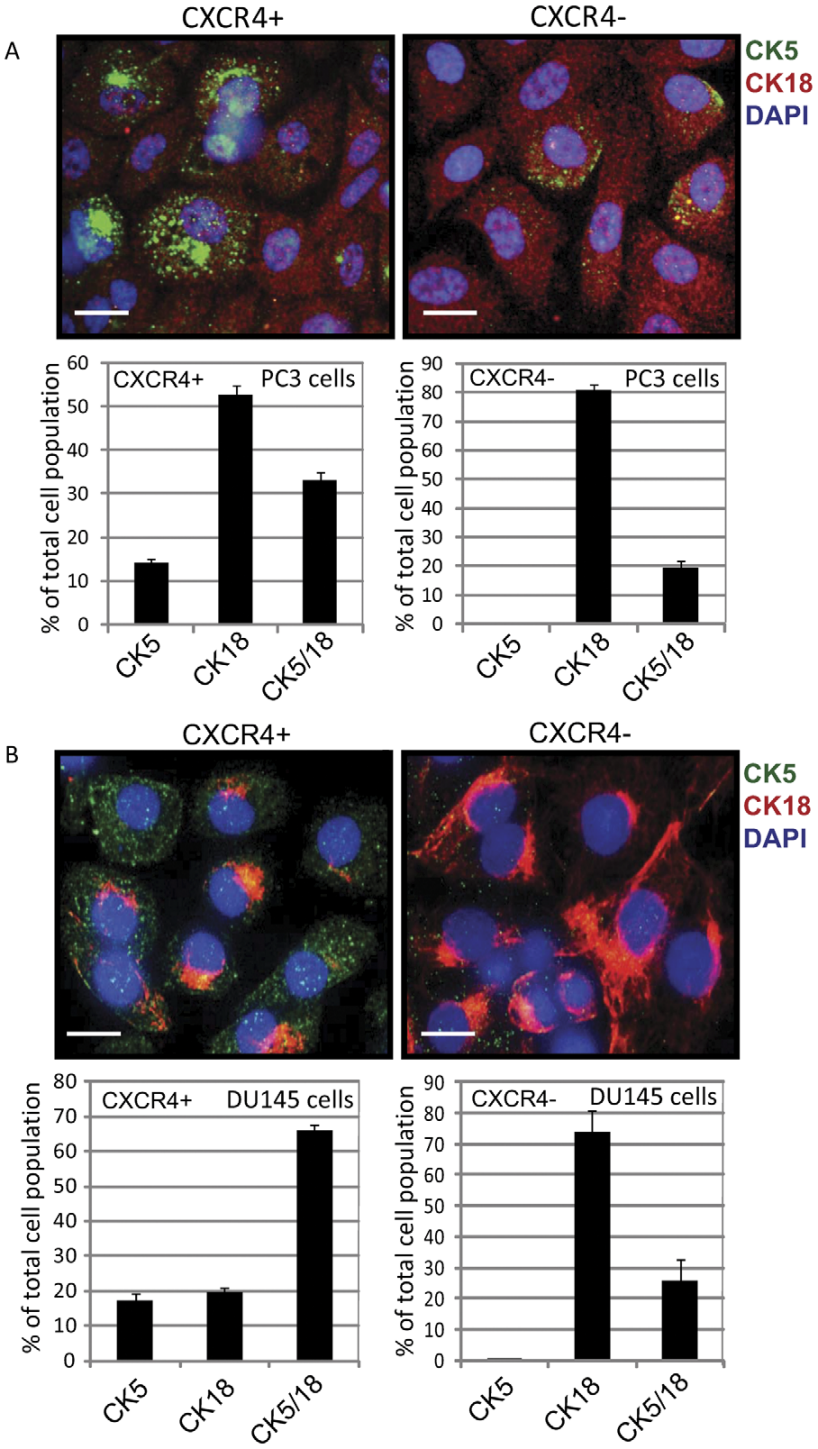
CXCR4^+^ populations in DU145 and PC3 cell lines are multipotent. FACS purified CXCR4^+^ and CXCR4^−^ DU145 and PC3 cells were cultured under differentiation conditions in the presence of 10% FBS.CXCR4^+^ cells differentiate toCK5^+^ basal epithelial cells (10.7%), CK5^+^/CK18^+^ intermediate cells (32.2%), and CK18^+^ luminal epithelial cells (57.1%). In contrast, CXCR4^−^ cells differentiate to CK18^+^cells (91.9% of total cell population) and CK5^+^/CK18^+^ cells (8% of total cell population) but not basal epithelial cells. Scale bars indicate 15 µm.

Finally, we determined whether CXCR4^+^ and CXCR4^−^ cells exhibit distinct differentiation potential by analyzing these cell populations for expression of prostate lineage markers. Previous studies demonstrated that prostate cancer can originate from CK5^+^ basal cells with multilineage differentiation potential [23], [24]. DU145 and PC3 cells were sorted into the CXCR4^+^ and CXCR^−^ fractions and grown on tissue culture treated plastic under differentiation conditions. Interestingly, CXCR4^+^ populations in DU145 and PC3 cell lines are more heterogeneous as compared to CXCR4^−^ cells and include a CK5^+^ population of basal epithelial cells, intermediate population of basal-like CK5^+^/CK18^+^ cells, and CK18^+^ population of luminal epithelial cells. In contrast, CXCR4^−^ cells differentiate to CK5^−^/CK18^+^ and CK5^+^/CK18^+^ cells but do not give rise to CK5^+^ basal epithelial cells (Figure 3). This result suggests that the CXCR4^+^ population harbors more tumorigenic basal-like cells, which is consistent with recent findings that basal epithelial cells are a cell of origin for prostate cancer [25].

### CXCR4/CXCL12 pathway regulates prostate cancer cells through a PI3K/AKT/FOXO3A dependent feedback loop

**Figure 4:**
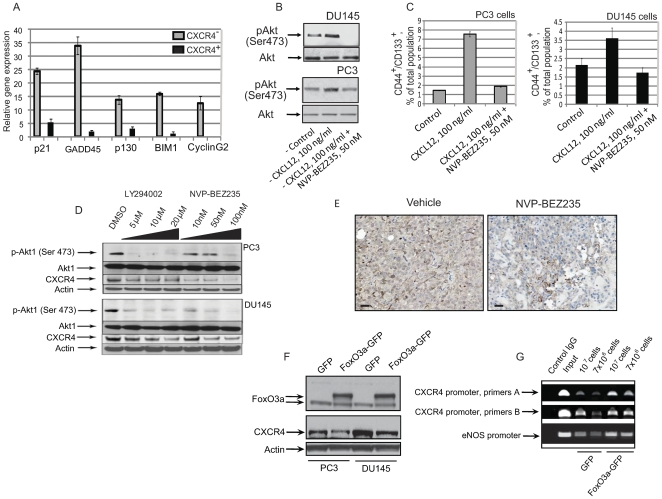
The PI3K pathway mediates the function of CXCR4 in prostate tumor initiating population. (A) Isolated CXCR4^+^ PC3 cells showed decreased expression levels of FOXO3A responsive genes such as p21, GADD45, p130, BIM1, CyclinG2 compared to CXCR4^−^PC3 cells. mRNA levels were determined by quantitative RT-PCR and normalized to GAPDH mRNA. (B) Treatment of prostate cancer cells with CXCL12 induced activation of the PI3K/AKT pathway. DU145 and PC3 cells were grown in serum-free, EBM medium with supplements and treated with the indicated concentrations of CXCL12 and NVP-BEZ235 for 18 hours. (C) Proliferative effect of CXCR4/CXCL12 signaling on CD44^+^/CD133^+^ progenitors could be completely abolished by PI3K inhibition. DU145 and PC3 cells were grown in serum-free, EBM medium with supplements and treated with the indicated concentrations of CXCL12 and NVP-BEZ235 replenished daily for 5 days. (D) Treatment of DU145 and PC3 cells with PI3K inhibitors LY294002 and NVP-BEZ235 reduced the level of CXCR4. (E) PI3K inhibition decreases CXCR4 expression *in vivo*. DU145 cells were injected subcutaneously into NOD/SCID mice. When the tumors had grown to a size of 300 mm^3^, the mice were treated with NVP-BEZ235 (12.5 mg/kg given every day perorally) or with vehicle for 5 weeks. Histological analysis of paraffin embedded sections demonstrates reduction of CXCR4 expression in xenograft tumors treated with the PI3K inhibitor. Scale bars indicate 15 µm. (F) DU145 cells stably transfected with FOXO3A-GFP have decreased CXCR4 expression. Western blot analysis shows endogenous and overexpressed FOXO3A fusion proteins (arrows). (G) ChIP assay demonstrates that FOXO3A binds to the CXCR4 promoter. DU145 and PC3 cells were stably transfected with either GFP or FOXO3A-GFP as described [17]. Cells were crosslinked with formaldehyde and chromatin was immunoprecipitated with anti-FOXO3A/FKHRL1 antibody. The crosslinked genomic CXCR4 promoter was detected by PCR. FOXO3a regulated gene eNOS was used as a positive control. The amount of precipitated FOXO3A-CXCR4 promoter was increased in DU145 cells stably transfected with FOXO3A-GFP protein.

Recently we showed that activation of the PI3K/AKT pathway is important for prostate cancer progenitor self-renewal and tumorigenicity [5], [12]. Moreover, a previous study demonstrated that the CXCR4/CXCL12 interaction activates PI3K/AKT signaling in prostate cancer cells [26]. Thus CXCR4 may contribute to maintenance of prostate cancer progenitors through activation of the PI3K/AKT axis. PI3K/AKT signaling regulates transcription through the forkhead family of transcription factors (FOXO) by phosphorylating conserved serine/threonine residues. Transcriptionally active FOXOs affect a wide range of biological processes, including cell survival, DNA repair, oxidative stress response, and longevity [27]. Among the members of the FOXO family, FOXO3A has been shown to be important for the maintenance of neural, hematopoietic, and endothelial stem cells [28], [29], [30], and prostate cancer stem-like cell populations [5], [12]. Consistent with earlier experiments which showed that FOXO3a-dependent gene expression is inhibited in the CD44^+^/CD133^+^ prostate cancer progenitors versus CD44^−^/CD133^−^ cells [5], we found that the FACS purified CXCR4^+^ PC3 cell population showed decreased expression levels of FOXO3A responsive genes such as p21, GADD45, p130, BIM1, and CyclinG2 compared to CXCR4^−^ cells, suggesting that increased expression of CXCR4 is associated with PI3K activation (Figure 4A). Treatment of prostate cancer cells with CXCL12 at a concentration of 100 ng/ml induced activation of the PI3K/AKT pathway (up to 1.5–2.0 fold increase in AKT phosphorylation in PC3 and DU145 cells, respectively) (Figure 4B), in addition to increasing the CD44^+^/CD133^+^ population for both PC3 and DU145 cells (Figure 2D). Conversely, the PI3K inhibition completely abolished the effect of CXCL12 on the proliferation of CD44^+^/CD133^+^ progenitors (Figure 4C), and reduced the level of CXCR4 expression in both DU145 and PC3 cells *in vitro* and *in vivo* (Figure 4D, E).

We also observed a decrease in CXCR4 protein levels in response to FOXO3A overexpression in DU145 cells (Figure 4F) suggesting that FOXO3A could regulate CXCR4 levels directly through CXCR4 transcriptional regulation or indirectly via a different FOXO3A target gene. Inspection of the CXCR4 promoter [31] revealed a putative FOXO binding sequence GCAAACA [32] from positions −187 to −181. In order to confirm that FOXO3A binds to the CXCR4 promoter, a chromatin immunoprecipitation assay (ChIP) was performed. The amount of precipitated CXCR4 promoter was increased in DU145 cells stably transfected with FOXO3A-GFP protein (Figure 4G) demonstrating that FOXO3A binds to CXCR4 promoter. These data show a relationship between PI3K/AKT signaling activation, CXCR4 expression and the self renewal capacity and tumorigenicity of CD44^+^/CD133^+^ prostate cancer progenitor cells.

### CXCR4 regulates tumor progenitor cell adhesion

**Figure 5:**
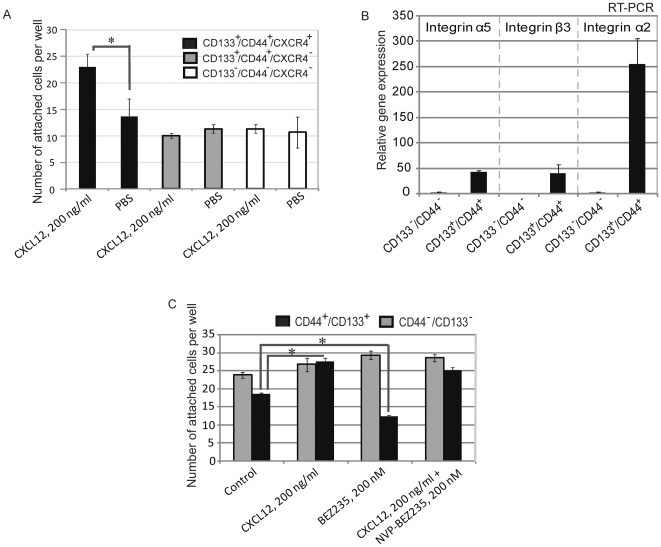
CXCR4 regulates tumor progenitor cell adhesion. (A) DU145 CD44^+^/CD133^+^/CXCR4^+^ cells have significantly higher CXCL12-dependent adhesion to fibronectin than CD44^+^/CD133^+^/CXCR4^−^ or CD44^−^/CD133^−^/CXCR4^−^ cells populations. Cells were FACS sorted and plated on fibronectin coated plates and allowed to adhere for 1 h. The cells were counted in three wells per condition using a phase-contrast microscope.*-p value<0.05. (B) The expression level of α2, α5, and β3 integrins subunits is strongly upregulated in CD133^+^/CD44^+^ DU45 compared to CD133^−^/CD44^−^ DU45 cells. (C) Inactivation of the PI3K pathway with NVP-BEZ235 significantly decreases CXCR4-dependent adhesion of CD133^+^/CD44^+^ DU45 cells to fibronectin. The adhesion of CD133^+^/CD44^+^ DU45 cells could be restored in the presence of CXCL12. The adhesion of CD133^−^/CD44^−^ DU45 did not respond to the CXCR4 and PI3K signaling modulation, *-p value<0.05.

The role of CXCR4 in cancer progression has generally been analyzed in the context of cancer cell metastasis [19], [20], [21], [22], [33]. At the molecular level, CXCR4 is an important mediator of the interaction of prostate tumor cells with extracellular matrix proteins such as laminin, fibronectin, and collagen which contributes to the metastatic process [14], [34], [35]. Despite the fact that CXCR4 does not directly modulate cell attachment, CXCR4 receptor engagement by CXCL12 plays an essential role in managing cell adhesion by modulation of integrin expression, FAK phosphorylation, and activation of p38 MAPK and ROCK kinases [34], [36]. To examine the role of CXCR4 in the cell adhesion and migration of prostate tumor initiating cells, we compared FACS sorted CD44^+^/CD133^+^/CXCR4^+^ cells with other populations in cell adhesion assays. We found that CD44^+^/CD133^+^/CXCR4^+^ cells have significantly higher CXCL12-dependent adhesion to fibronectin than CD44^+^/CD133^+^/CXCR4^−^ or CD44^−^/CD133^−^/CXCR4^−^ cells populations (Figure 5A). The CXCL12-induced adhesion of prostate cancer cells to the extracellular matrix is mediated by integrins. Interestingly, the expression levels of α2, α5, and β3 integrin subunits are strongly upregulated in CD133^+^/CD44^+^ DU145 compared to CD133^−^/CD44^−^ DU145 cells (Figure 5B), consistent with recent studies which show that activation of the CXCR4/CXCL12 pathway in DU145 cells leads to enhanced expression of α5 and β3 integrins [14]. Additionally, inactivation of the PI3K pathway with NVP-BEZ235 (200 nM) significantly decreased CXCR4-dependent adhesion of CD133^+^/CD44^+^ DU145 cells to fibronectin, and this inhibition could be abolished in the presence of 200 ng/mL CXCL12 (Figure 5C). Remarkably, adhesion of CD133^−^/CD44^−^ DU145 cells did not respond to CXCL12 treatment or PI3K signaling inhibition (Figure 5C). Collectively, these studies suggest that expression of CXCR4 could provide a selective advantage for interaction with the extracellular matrix and facilitate preinvasive binding of the tumor progenitor cells allowing further tumor dissemination.

### Inhibition of the CXCR4 pathway leads to a decrease in prostate cancer progenitor populations

**Figure 6:**
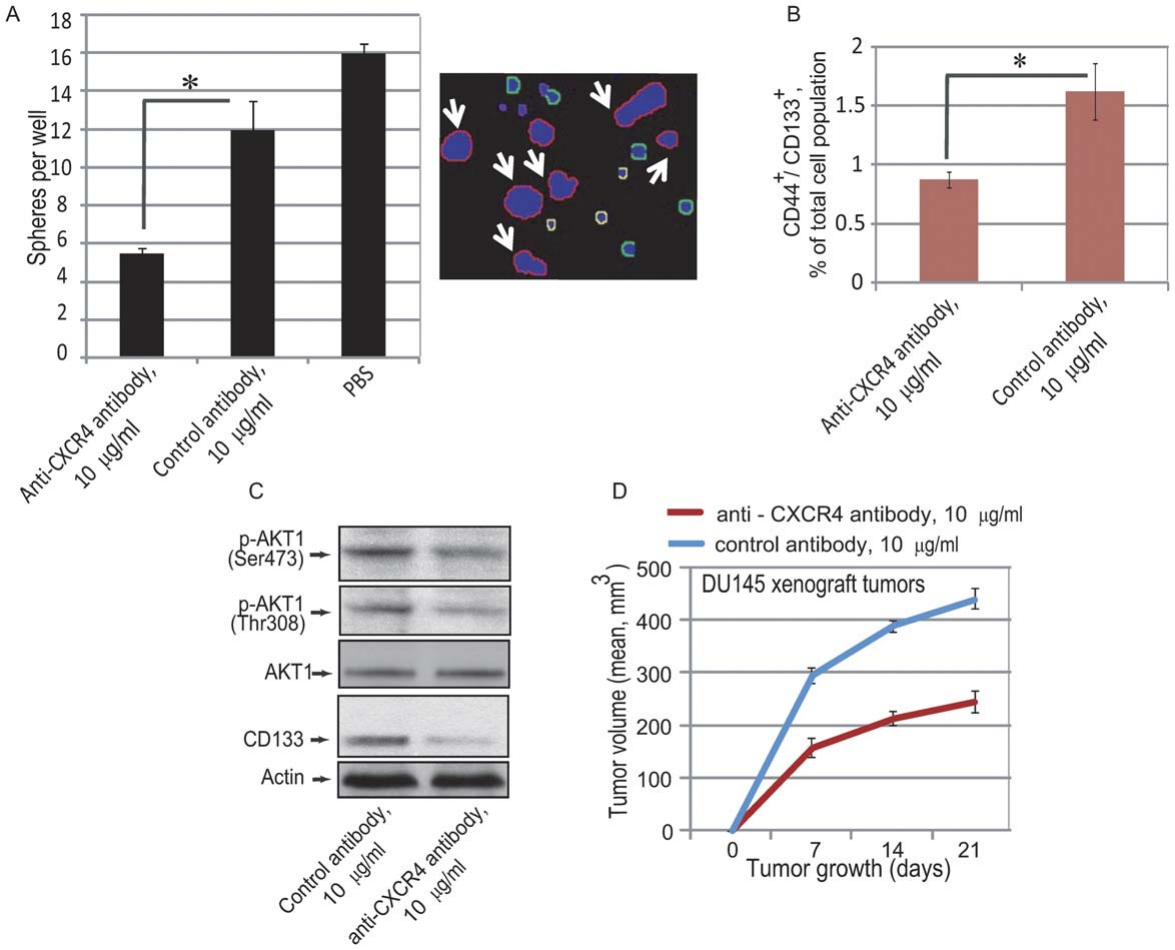
CXCR4 neutralization leads to attenuation of the CD44^+^/CD133^+^ prostate progenitor population. (A) DU145 cells were plated in 96-well low-attachment plates at 100 cells per well (5 replicates) and the spheres were grown in serum-free, EBM medium with supplements. The antibodies were replenished daily. Cells were imaged with an Acumen eX3 microplate cytometer and spheres were detected using image analysis software. The sphere size was measured by GFP intensity. The spheres were discriminated from cell debris using a Gaussian filter. The spheres included in the analysis are outlined in red and indicated by arrows. Representative data from one of two independent experiments is shown; *- p value<0.05. (B) Flow cytometry analysis revealed attenuation of CD44^+^/CD133^+^ population in DU145 cells treated with 10 µg/ml neutralizing anti-CXCR4 (mouse monoclonal IgG, clone 44716, R&D Systems) or 10 µg/ml control antibody (mouse IgG isotype control, Lifespan Bioscience Inc.) for 5 days. The cell were grown in medium supplemented with 2% FBS. Culture medium was refreshed every second day; *- p value<0.05. (C) Western blot analysis of DU145 cells treated with 10 µg/ml neutralizing anti-CXCR4 antibody for 5 days demonstrated downregulation of the PI3K/AKT pathway activation compared to the cells treated with 10 µg/ml control antibody. The cell were grown in medium supplemented with 2% FBS. Culture medium was refreshed every second day. (D) Preincubation of prostate cancer cells with neutralizing anti-CXCR4 antibody significantly delays tumor growth. 5×10^5^ DU145 cells pretreated with neutralizing anti-CXCR4 or control antibody for 5 days were embedded in BD matrigel and injected s.c. into NOD/SCID mice.*- p value<0.01.

To provide additional evidence that the CXCR4/CXCL12 signaling is important for stem-like cell maintenance and targeting of this pathway can lead to inhibition of prostate cancer progenitor growth, we tested if inactivation of the CXCR4/CXCL12 axis by a neutralizing antibody affects prostate cancer progenitors *in vitro* and *in vivo*. DU145 cells were treated with 10 µg/mL neutralizing anti-CXCR4 (mouse monoclonal IgG, clone 44716, R&D Systems) or control antibody (mouse IgG isotype control, Lifespan Bioscience Inc.) for 5 days in serum-free epithelial growth medium and sphere forming ability was measured. Treatment with anti-CXCR4 antibody leads to a 2.2-fold decrease in sphere forming ability in DU145 cells compared to cells treated with control antibody (Figure 6A). Furthermore, flow cytometry analysis revealed a more than 2-fold decrease in the CD44^+^/CD133^+^ population in DU145 cells pretreated with anti-CXCR4 antibody (Figure 6B) as well as inhibition of the PI3K/AKT1 signaling pathway (Figure 6C). Preincubation of DU145 cells with anti-CXCR4 antibody before subcutaneous injection into NOD/SCID mice significantly delayed tumor growth (Figure 6D), compared to cells treated with control antibody.

**Figure 7:**
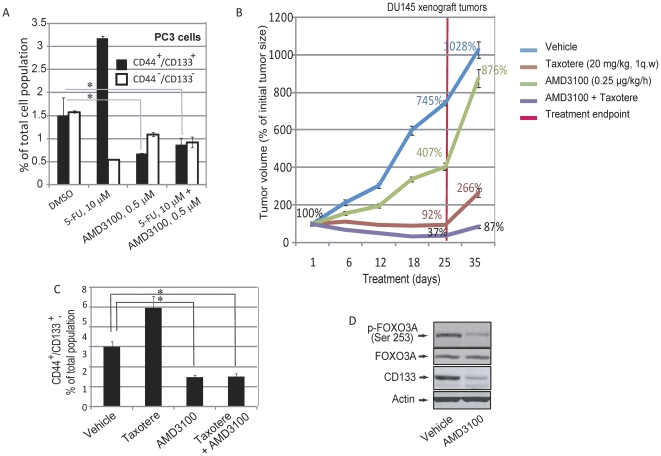
Targeting the tumor initiating and differentiated populations within DU145 and PC3 carcinoma cell lines by CXCR4 antagonist and conventional therapy. (A) Treatment with CXCR4 antagonist AMD3100 decreases the CD133^+^/CD44^+^ population but did not significantly affect the CD133^−^/CD44^−^ population within prostate cancer cells. The use of cytotoxic drugs alone results in a decrease in proliferating tumor cells, but leads to an overall increase in the relative population of tumor initiating cells. Combinatorial treatment of PC3 cells with AMD3100 and the conventional chemotherapeutic drug 5-fluouracil decreases both undifferentiated and differentiated cells populations. PC3 cells were grown in serum-free, EBM medium with supplements and treated with the indicated concentrations of AMD3100 and 5-fluouracil. On the 5^th^ day the cells were subjected to flow cytometry analysis; *-p value<0.05. (B) Combinatorial therapy *in vivo* demonstrates significant inhibition of tumor growth compared to single drug treatment. The mice were treated with Taxotere (20 mg/kg, 1q.w., p.o) as described (19). Alzet pumps were used to deliver AMD3100 at a constant rate of 0.25 µg/kg/hour. The pumps loaded with AMD3100 or saline were implanted subcutaneously. The mice were observed for 8 weeks for appearance and development of tumors. (C) CD133 and CD44 immunostaining on frozen sections of xenograft tumors treated with combinatorial or mono-therapy from (B) revealed selective inhibition of the CD133^+^/CD44^+^ population by the CXCR4 antagonist AMD3100; *-p value<0.05. (D) Western blot analysis showed a significant decrease in FOXO3A phosphorylation in the tumors treated with AMD3100.

We then tested the effects of the CXCR4-specific small molecule antagonist AMD3100 on the survival of the progenitor population (CD44^+^/CD133^+^) within prostate cancer cell lines. PC3 cells were treated with 0.5 µM AMD3100 or with 75 µM of the conventional chemotherapeutic drug 5-fluorouracil for 4 days in serum-free epithelial growth medium. Flow cytometry analysis revealed a 2.2-fold decrease in the CD44^+^/CD133^+^ population with AMD3100 treatment (Figure 7A and Figure S2A), whereas this population was increased up to 2.1 fold in response to conventional therapy. In contrast, the CD44^−^/CD133^−^ population was sensitive to conventional chemotherapy showing a 2.1-fold decrease, but was unresponsive to AMD3100 treatment. Additionally, the combination of AMD3100 and the chemotherapeutic drug resulted in a simultaneous decrease of both CD44^+^/CD133^+^ and CD44^−^/CD133^−^ cell populations (≥1.8-fold decrease and 1.7-fold decrease respectively).

### Combination therapy targeting progenitor and bulk tumor cells leads to enhanced tumor regression

To determine whether the *in vitro* effect seen with the combination of AMD3100 and 5-fluorouracilcan be recapitulated *in vivo*, DU145 hormone-refractory prostate carcinoma xenograft tumors were treated with a combination of the AMD3100 and the cytotoxic antimicrotubule agent Taxotere. 500,000 DU145 cells were injected subcutaneously into NOD/SCID mice. When the tumors had grown to a size of 300 mm^3^, the mice were treated with Taxotere (20 mg/kg, injected once-weekly intravenously), AMD3100 (given in subcutaneous osmotic pumps loaded with 20 mg/mL AMD3100 with an infusion rate 0.5 µl/h), or the combination. After 25 days, the xenograft tumors treated with AMD3100 or Taxotere alone showed a decrease in tumor growth rate compared to the control group (a 1.8-fold decrease and 8-fold decrease, respectively) (Figure 7B). The combination treatment, on the other hand, led to a 20-fold difference in tumor size relative to the control, starting at week two. Histological analysis revealed more than 2-fold attenuation of the CD44^+^/CD133^+^ population in the xenograft tumors treated with AMD3100, alone or in combination with Taxotere. In contrast, the CD44^+^/CD133^+^ population was increased after Taxotere monotherapy (Figure 7C and Figure S2B). Western blot analysis showed a decrease in FOXO3A phosphorylation in tumors treated with AMD3100 alone, suggesting that enhanced tumor regression upon treatment with AMD3100 is mediated by inhibition of the PTEN/PI3K/AKT pathway (Figure 7D). We observed that the tumors treated with both Taxotere and NVP-BEZ235 showed slower regrowth during the 10 days after the treatment was terminated as compared with tumors treated with Taxotere alone (a 2.3-fold increase and 2.9-fold increase, respectively) (Figure 7B). The endpoint tumors treated with combination therapy were still a modest 210 mm^3^ on average compared to nearly 480 mm^3^ for Taxotere alone suggesting that treatment with CXCR4 antagonists directed against progenitor cells may be useful in combination with conventional drugs in prostate cancer treatment. Taken together, these studies suggest that targeting the CXCR4/CXCL12 pathway results in decreased prostate progenitor survival *in vitro* and *in vivo* in androgen refractory cancer cell lines and potentially could be of therapeutic value against advanced prostate cancers.

## Discussion

A growing body of evidence has demonstrated that many human tumors contain a heterogeneous mixture of different cell types and are maintained by subpopulations of tumor progenitors [4]. Tumor progenitor cells can divide symmetrically to self-renew and asymmetrically to differentiate into heterogeneous tumors and cause tumor formation and subsequent metastasis. Prostate cancer progenitor populations can be defined by the expression of cell surface markers CD44, CD133, and α2β1^high^ integrin [5], [7], [8], [9], [10], [11], [12]. Whereas prostate cancer cell lines have less than a 2% progenitor population when cultured under long-term monolayer culture conditions, prostate cancer progenitor populations expressing CD44 and CD133 cell surface markers can be significantly enriched when grown under sphere-forming conditions [5], [11]. Effective targeting of the tumor initiating cell population requires a detailed understanding of the cellular pathways that contribute to maintenance of stemness. To better understand self-renewal pathways in prostate cancer progenitors, we performed gene expression profiling and found that CXCR4 is highly expressed in prostate cancer cells grown as spheres. This was an intriguing finding because of CXCR4's well-known role in tumor metastasis. While the role of CXCR4 has been examined in the cancer stem cell context in pancreatic cancer [33], characterization of CXCR4 signaling in prostate cancer progenitors has not been reported.

We confirmed increased expression of CXCR4 in prostate cancer cells grown under sphere forming conditions compared to monolayer conditions, and in CD44^+^/CD133^+^ prostate tumor initiating cells compared to CD44^−^/CD133^−^ cells. Further, the relationship between CXCR4 and tumor initiating cell markers CD133/CD44 is reciprocal: CXCR4^+^ cells have a higher percentage of CD133^+^/CD44^+^ than CXCR4^−^ cells and CD133^+^/CD44^+^ cells contain more CXCR4^+^ cells than CD133^−^/CD44^−^ cells. This finding implies that in prostate tumor initiating cells, CXCR4 is important for the maintenance of stemness. In contrast to pancreatic cancer stem cells in which CD133^+^/CXCR4^−^ cells are just as tumorigenic as CD133^+^/CXCR4^+^ cells, in prostate tumor initiating cells CXCR4 expression results in significantly greater colony formation (Figure 2F) and inhibition of CXCR4 with an antagonistic antibody reduces tumor growth (Figure 6D). In addition, our previous work showed that the PI3K/AKT/FOXO3A axis is a critical regulator of CD44^+^/CD133^+^ progenitor populations within prostate cancer cells, and that FOXO3a-dependent gene expression is inhibited in CD44^+^/CD133^+^ prostate cancer progenitors versus CD44^+^/CD133^+^ cell population. Our current work suggests that the CXCL4/CXCR12 signaling pathway may be an upstream regulator of the PI3K signaling and thus plays multiple roles in prostate cancer disease progression. Interestingly, we found that inhibition of PI3K signaling leads to a reduction in CXCR4 expression. Chromatin-IP with FOXO3A as a target showed a direct physical interaction between FOXO3A and the CXCR4 promoter. To our knowledge this is the first report of direct regulation by the PI3K pathway of CXCR4 expression and thus describes a mutually positive regulatory feedback loop between the PI3K/AKT and CXCR4/CXCL12 signaling pathways which are both important for tumor initiating cell self renewal.

CXCR4 is one of the key regulators of tumor invasiveness and metastasis development [13], [15], [16], [17], [18], [19], [20], [21], [22]. Blocking CXCR4 receptor function by a monoclonal antibody inhibits cancer cell proliferation, motility and invasion in multiple preclinical models both *in vitro* and *in vivo* [22], [37]. Expression of CXCR4 is linked to the tendency of prostate cancer cells to metastasize to the bone, a tissue that expresses a high level of the chemokine CXCL12 [25], [38]. Our data suggest that CXCL12 regulates the adhesion of CD133^+^/CD44^+^ prostate cancer progenitors to the extracellular protein fibrionectin which is important for distal organ seeding and initiation of secondary tumors. Moreover, the expression of α5, and β3 integrin subunits, which form receptors for fibronectin, are strongly upregulated in CD133^+^/CD44^+^ DU145 compared to CD133^−^/CD44^−^ DU145 cells, consistent with a recent study which demonstrated enhanced expression of α5 and β3 integrins in DU145 cells in response to activation of the CXCR4/CXCL12 pathway [14]. Fibronectin receptor α_V_β_3_ has been found to facilitate prostate cancer metastasis to bone by mediating prostate cancer cell adhesion. It is noteworthy that α_V_β_3_ dependent prostatic carcinoma cell migration requires the activation of focal adhesion kinase and the subsequent activation of the PI3K/AKT signaling pathway [39], [40].

Taken together, our studies have revealed additional components of prostate cancer stem cell signaling (Figure S3). CXCL12 is an important input in CXCR4^+^ prostate tumor initiating cells and leads to an elevated activation of the PI3K pathway and more robust proliferation of prostate cancer progenitors. Increased CXCR4 expression also leads to greater integrin-mediated adhesion of cells to extracellular matrix substrates. These findings suggest that inhibition of the CXCR4/CXCL12 pathway in prostate cancer progenitors could lead to more effective cancer treatment and may provide synergistic antitumor activity with conventional therapy. We observed that inhibition of CXCR4 by the small molecule antagonist AMD3100 or blockage of CXCR4/CXCL12 interaction by a neutralizing anti-CXCR4 antibody could specifically inhibits proliferation of prostate progenitor population in PC3 and DU145 prostate cancer cell lines *in vitro* and *in vivo* and may provide a new approach to deplete prostate cancer stem cells. Other experiments have also shown that CXCR4 antagonist CTCE-9908 reduced growth of prostate xenografts via inhibition of angiogenesis and lymphangiogenesis, and induction of apoptosis [41].

In summary, our studies showed that similar to leukemia and breast cancer, several progenitor cell-like subpopulations can exist in prostate cancer [42], [43], [44]. Cancer stem cells are likely hierarchical populations playing different roles in cancer initiation and progression (Figure S4). CD133^+^/CD44^+^/CXCR4^+^ cells represent a highly tumorigenic subset of cancer progenitors and targeting CXCR4 signaling may be beneficial in eliminating this subpopulation.

## Materials and Methods

### Cells, reagents and animals

DU145 and PC3 prostate cancer cell lines were obtained from the ATCC and cultured in the recommended medium containing 10% FBS. NOD.CB17-Prkdc (SCID) mice were obtained from Jackson Laboratories and maintained under standard conditions according to institutional guidelines. All surgery was performed under sodium pentobarbital anesthesia, and all efforts were made to minimize suffering. The antibodies used were:anti-CXCR4 (ab2074 (Abcam) and MAB172 (R&D Systems)); anti-β-actin (mAb, Sigma); anti-CD133 (directly conjugated with phycoerythrin (PE), clone 293C3, Miltenyi Biotech Ltd); anti-CD44v6 (directly conjugated with allophycocyanin (APC), clone C44-26, BD Pharmingen); anti-Cytokeratin 5 (clone RCK103, Santa-Cruz),anti-Cytokeratin 18 (clone E431-1, Epitomics), donkey anti-rabbit and sheep anti-mouse IgG, HRP-linked whole Ab(GE Healthcare). CXCR4 antagonist AMD3100 was purchased from Sigma Aldrich (St. Louis, MO). Taxotere was purchased from LC Laboratories (Woburn, MA).

### Colony formation assay

Cells were plated in 12 well plates at 500 or 1000 cells per well in triplicate and grown in medium containing 10% FBS for 10 days. The cell were fixed with 10% formalin for 30 min and stained with 0.05% crystal violet in distilled water for 30 min, then washed twice with distilled water and air dried.

### Sphere formation assay

Single cells were plated at 100, 500 or 1000 cells/mL per well in triplicates in 24 well low-attachment plates with ultra-low attachment surface (Fisher Scientific Co., Pittsburgh, PA). Cells were grown in serum-free Epithelial Basal Medium (EBM, with bicarbonate and phenol red; Cambrex) supplemented with 4 µg/mL insulin (Sigma), B27, 20 ng/mL epidermal growth factor EGF, 20 ng/mL basic fibroblast growth factor FGF (Invitrogen, Carlsbad, CA). Spheres were analyzed after 7 to 14 days.

### Attachment assay

384 well black clear bottom plates (Greiner Bio-One) were coated with fibronectin (diluted to 50 µg/ml in PBS, Sigma) overnight. Plates were washed with 1% bovine serum albumin (BSA) in PBS to block nonspecific cell adhesion. DU145 cells were plated at a density of 100 cells per well in serum-free Epithelial Basal Medium (EBM, with bicarbonate and phenol red; Cambrex) supplemented with 4 µg/mL insulin (Sigma), B27, 20 ng/mL epidermal growth factor EGF, 20 ng/mL basic fibroblast growth factor FGF (Invitrogen, Carlsbad, CA). The cells were allowed to adhere for 1 h. Subsequently, the cells were fixed with 3.7% formaldehyde in PBS. Non-adherent cells were washed off and the remaining cells were counted with a microscope.

### Flow cytometry analysis of CXCR4, CD44 and CD133 expression

For flow cytometry, cells were dissociated with Accutase (Innovative Cell Tech Inc., San Diego, CA, USA) and washed 2 times in staining solution containing Ca^2+^ and Mg^2+^-free PBS with 1 mM ethylenediaminetetraacetic acid (EDTA), 25 mM Hepes (pH 7.0) (Gibco BRL), and 1% FBS. Cells were stained live in staining solution containing conjugated anti-CD44 and anti-CD133 antibody for 50 min at 4°C. For CXCR4 staining, the cells were incubated with unconjugated anti-CXCR4 antibody (MAB172; R&D Systems) followed by staining with anti-mouse secondary antibody conjugated with Alexa 488. Samples were analyzed on a BD LSR II flow cytometer (Beckton Dickinson Immunocytometry Systems, San Jose, CA). A minimum of 500,000 viable cell events were collected per sample. For sorting, 2×10^7^ cells were processed for CD44 and CD133 multi-color staining along with appropriate negative controls and single color positive controls. The CD44^+^/CD133^+^ and CD44^−^/CD133^−^ populations were sorted on a BD FACS Diva cell sorter (Beckton Dickinson Immunocytometry Systems, San Jose, CA).

### Histology and immunofluorescence

For cryosectioning, the tumors were fixed by immersion in 4% paraformaldehyde, cryoprotected in 20% sucrose, frozen, and embedded in sucrose∶OCT (1∶1). Cryostat sections (12 µm) were collected on Superfrost plus slides. Slides were preincubated 30 min in antibody buffer (50 mM NaCl, 50 mM Tris Base, 1% BSA, 100 mM L-Lysine, 0.04% sodium azide [pH 7.4]) containing 0.4% Triton and 10% serum and then incubated overnight at 4°C with the primary antibodies anti-CD133 (Abcam, ab27699, dilution 1∶200) and anti-CD44 (Abcam, ab24504, dilution 1∶100). Bound antibodies were detected with appropriate secondary antibodies conjugated with Alexa 488 or 555 (1∶1000; Molecular Probes) diluted in antibody buffer at room temperature for 1 h. For CD133 detection, sections were incubated with a goat anti-mouse biotinylated secondary antibody (1∶400; Jackson Immunoresearch) followed by incubation with Avidin-Biotin Complex (ABC elite; Vector laboratories) for 30 min. The slides were then incubated with Streptavidin-FITC (Vector Laboratories). For quantification, cells in at least four randomly selected fields of view were counted for each condition. At least 1000 cells per condition were counted. For immunofluorescent microscopy, DU145 cells were plated in a 384 well black clear bottom plate (Greiner Bio-One) at a density of 100 cells/well in medium containing 10% serum. After 7 days, the cells were fixed for 30 min in 3.7% formaldehyde at room temperature and permeabilized with 0.125% Triton X-100 for 10 min. The cells were washed with PBS, and blocked by incubation with 10% BSA in PBS. The cells were then incubated with primary antibody diluted in 3% BSA in PBS (anti-Cytokeratin 5 (clone RCK103, Santa-Cruz, dilution 1/50) and anti-Cytokeratin 18 (clone E431-1, Epitomics, dilution 1/100) for 1 h and washed ten times with PBS. Cells were then incubated for 1 h with a secondary antibody conjugated with Alexa 488 or 555 (Invitrogen) diluted 1/500 in 3% BSA in PBS. After extensive washes with PBS the cells were stained with DAPI and examined under epifluorescent illumination. For quantification, 100 to 300 cells per condition were counted. For paraffin sectioning, tumor tissues were fixed in 10% Neutral Buffered Formalin (NBF). After 48 hour the tissues were processed with a mouse tissue processing cycle and then embedded in paraffin. Every 10^th^ slide was stained with Mayer's Hematoxylin and Eosin; the adjacent slides were stained with anti-CD133 and anti-CXCR4 antibodies. Immunohistochemistry was performed on a Ventana Discover XT. The slides were blocked with avidin/biotin and stained with anti-CD133 antibody for 1 hour (Abcam, ab19898 dilution 1∶100) with using Ventana CC1 HIER followed by incubation with biotinylated goat anti-rabbit antibody (Jackson ImmunoResearch) and labeled using a Ventana DABMap kit. Alternatively, the slides were blocked with Ventana antibody block and stained with anti-CXCR4 antibody for 12 hours (Abcam, ab2074, dilution 1∶50) using Ventana CC2 HIER followed by incubation with Ventana's Umap anti-rabbit HRP conjugated antibody and Chromo Dab map detection kit.

### In vivo tumorigenicity assay

Protocol 08-223 was approved by the Institutional Animal Care and Use Committee of the Genomics Institute of the Novartis Research Foundation. DU145 xenograft tumors were established using early-passage cells and maintained in NOD.CB17-Prkdc (SCID) mice. For subcutaneous tumor development 100 µl of collagen-embedded cells were injected s.c. into 5–8 week old NOD.CB17-Prkdc (SCID) mice. Treatment began when tumors were 100 mm^3^ in size. The mice were treated with Taxotere (20 mg/kg, 1q.w., p.o) as described (12). To ensure consistent levels of the antagonist throughout the 4 week experimental period, we used osmotic Alzet pumps (Alza Corporation, Palo Alto, CA) to deliver AMD3100 at a constant rate of 0.25 µg/kg/hour. The pumps loaded with AMD3100 or saline were implanted subcutaneously. The mice were observed for 8 weeks for appearance and development of tumors.

### Microarray analysis

Microarray analysis was carried out as described earlier (5). Briefly, total RNA was isolated from cell pellets using the RNeasy kit (Qiagen, Valencia, CA, USA). Sample preparation for GeneChip analysis was carried out according to the protocol detailed by Affymetrix (Santa Clara, CA, USA). Briefly, first and second cDNA strands were synthesized; double stranded cDNA was *in vitro* transcribed using the Affymetrix 3′ amplification kit; and the resulting cRNA was purified, fragmented and hybridized to oligonucleotide arrays (Human Genome U133 Plus 2.0 Array, catalog number 900467, www.Affymetrix.com) representing over 47,000 transcripts. Arrays were processed using standard Affymetrix protocols. The Affymetrix Hybridization Control Kit and Poly-A RNA control kit were used for hybridization. Probe values from CEL files were condensed to probe sets using the gcRMA package from Bioconductor (www.bioconductor.org) and the R program (R Development Core Team (2004). R: A language and environment for statistical computing. R Foundation for Statistical Computing, Vienna, Austria. ISBN 3-900051-07-0, URL http://www.R-project.org). The dataset was unlogged and median scaled to a target intensity of 100. Primer sets used for microarray validation shown in Table S1. Data deposition: all data is MIAME compliant and that the raw data has been deposited in the Gene Expression Omnibus (GEO) database, www.ncbi.nlm.nih.gov/geo (accession no. GSE10832).

### Chromatin Immunoprecipitation Assay (ChIP)

DU145 cells were stably transfected with FOXO3A-GFP or control GFP expressing vectors, as described previously (5). ChIP assays were performed according to the manufacturer's protocol (Magna ChIP kit, Millipore). In brief, 7×10^6^ or 1×10^7^ cells were fixed by directly adding formaldehyde (37% stock, Sigma) to the medium to a final concentration of 1%. After 10 min, 2 M glycine stock was added (final concentration of 0.125 M glycine, room temperature, 5 min incubation, gentle mixing) to stop cross-linking. The cells were washed with PBS twice and collected using a cell scraper. After centrifugation, cells were resuspended in swelling buffer (0.1 M Tris pH 7.6, 10 mM KOAc and 15 mM MgOAc) for 20 min. The nuclei were released using a Dounce homogenizer (16 strokes). The pellets were collected, lysed, and the nuclear extracts were subjected to sonication on ice with a sonic dismembrator model 500 (Fisher Scientific). Immunoprecipitation was performed overnight at 4°C using 2.5 µg of antibody (Anti-FOXO3A/FKHRL1, Millipore). Immunoprecipitated chromatin was extracted using a chromatin IP DNA purification kit (Active Motif). Primer sets used for PCR of CXCR4 promoter region shown in Table S1.

### Statistical analysis

The results of soft agar colony formation assays, flow cytometry analysis, cell proliferation assays, and *in vivo* tumorigenicity assays were analyzed by paired *t*-test. A *p* value of <0.05 was regarded as statistically significant

## Supporting Information

Figure S1 **PC3 CXCR4^+^ cells have a higher tumorigenic potential in vivo as compared to PC3 CXCR4^−^ cells.** (A) Secondary spheres formation assay showed the self-renewal capacity of CXCR4^+^ PC3 cells. The primary spheres were dissociated and single cells were plated at 500 or 1000 cells/mL per well in triplicate in 24 well low-attachment plates and grown under sphere forming conditions for 7 days. (B) Adenovirus-mediated overexpression of CXCR4 in prostate cancer cells resulted in a more than 2.5-fold increase of CD44^+^/CD133^+^ population. DU145 cells were infected with adenovirus encoding CXCR4 under the control of the tet-off regulatory system, and with control adenovirus and analyzed by flow cytometry 4 days after infection. For CXCR4 staining, the cells were incubated with unconjugated anti-CXCR4 antibody (MAB172; R&D Systems) followed by staining with anti-mouse secondary antibody conjugated with Alexa 488. (C) 10^3^ CXCR4^+^ and CXCR4^−^ PC3 cells collected by FACS sorting were embedded in BD matrigel and injected s.c. into NOD/SCID mice. *-p value<0.05.
(TIF)

Figure S2 **Targeting the tumor initiating population within DU145 and PC3 carcinoma cell lines by CXCR4 antagonist.** (A) Treatment with CXCR4 antagonist AMD3100 decreases the CD133^+^/CD44^+^ population. PC3 cells were grown in serum-free, EBM medium with supplements and treated with 0.5 µM AMD3100. On the 5^th^ day the cells were subjected to flow cytometry analysis. (B) CD133 and CD44 immunostaining on frozen sections of xenograft tumors treated with combinatorial or mono-therapy revealed selective inhibition of the CD133^+^/CD44^+^ population by the CXCR4 antagonist AMD3100.
(TIF)

Figure S3 **Mechanism of CXCR4/CXCL12 dependent maintenance of prostate cancer progenitors.** CXCL4/CXCR12 – induced transactivation of receptor tyrosine kinases (EGFR, HER2, IGF-1R, FGFR, etc.) contributes to enhanced invasive signals and metastatic growth. The CXCL12-induced adhesion of prostate cancer progenitors to the extracellular matrix is mediated by integrins. PI3K pathway is one of the key mechanisms mediating the function of CXCR4 in prostate tumor initiating population. Targeting CXCR4 signaling with small molecule inhibitors may be beneficial in eliminating prostate cancer stem-like cells.
(TIF)

Figure S4 **Model of prostate cancer cell heterogeneity.** CXCR4^+^ cells represent a highly tumorigenic subset of cancer progenitors that could also have migratory properties.
(TIF)

Table S1 List of primers used for RT-PCR and ChIP assay.
(DOC)
