# Supplementary figures and images for: CXCR4 Expression in Prostate Cancer Progenitor Cells
Source: PLoS One. 2012 Feb 16;7(2):e31226. doi: 10.1371/journal.pone.0031226 (PMC3281066; doi:10.1371/journal.pone.0031226)

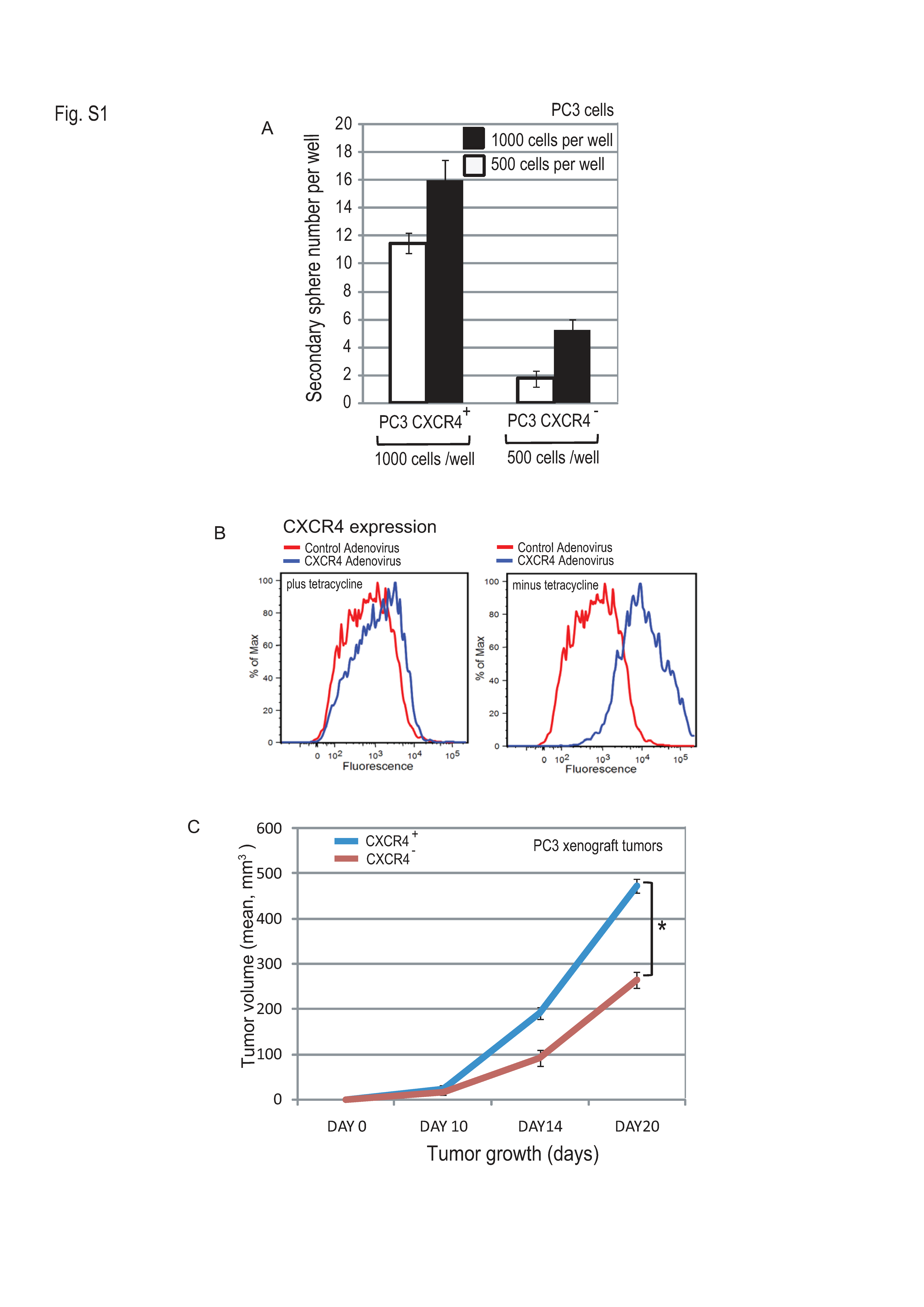

Supplement: Figure S1 — PC3 CXCR4+ cells have a higher tumorigenic potential in vivo as compared to PC3 CXCR4− cells. (A) Secondary spheres formation assay showed the self-renewal capacity of CXCR4+ PC3 cells. The primary spheres were dissociated and single cells were plated at 500 or 1000 cells/mL per well in triplicate in 24 well low-attachment plates and grown under sphere forming conditions for 7 days. (B) Adenovirus-mediated overexpression of CXCR4 in prostate cancer cells resulted in a more than 2.5-fold increase of CD44+/CD133+ population. DU145 cells were infected with adenovirus encoding CXCR4 under the control of the tet-off regulatory system, and with control adenovirus and analyzed by flow cytometry 4 days after infection. For CXCR4 staining, the cells were incubated with unconjugated anti-CXCR4 antibody (MAB172; R&D Systems) followed by staining with anti-mouse secondary antibody conjugated with Alexa 488. (C) 103 CXCR4+ and CXCR4− PC3 cells collected by FACS sorting were embedded in BD matrigel and injected s.c. into NOD/SCID mice. *-p value<0.05. (TIF) [file pone.0031226.s001.tif]

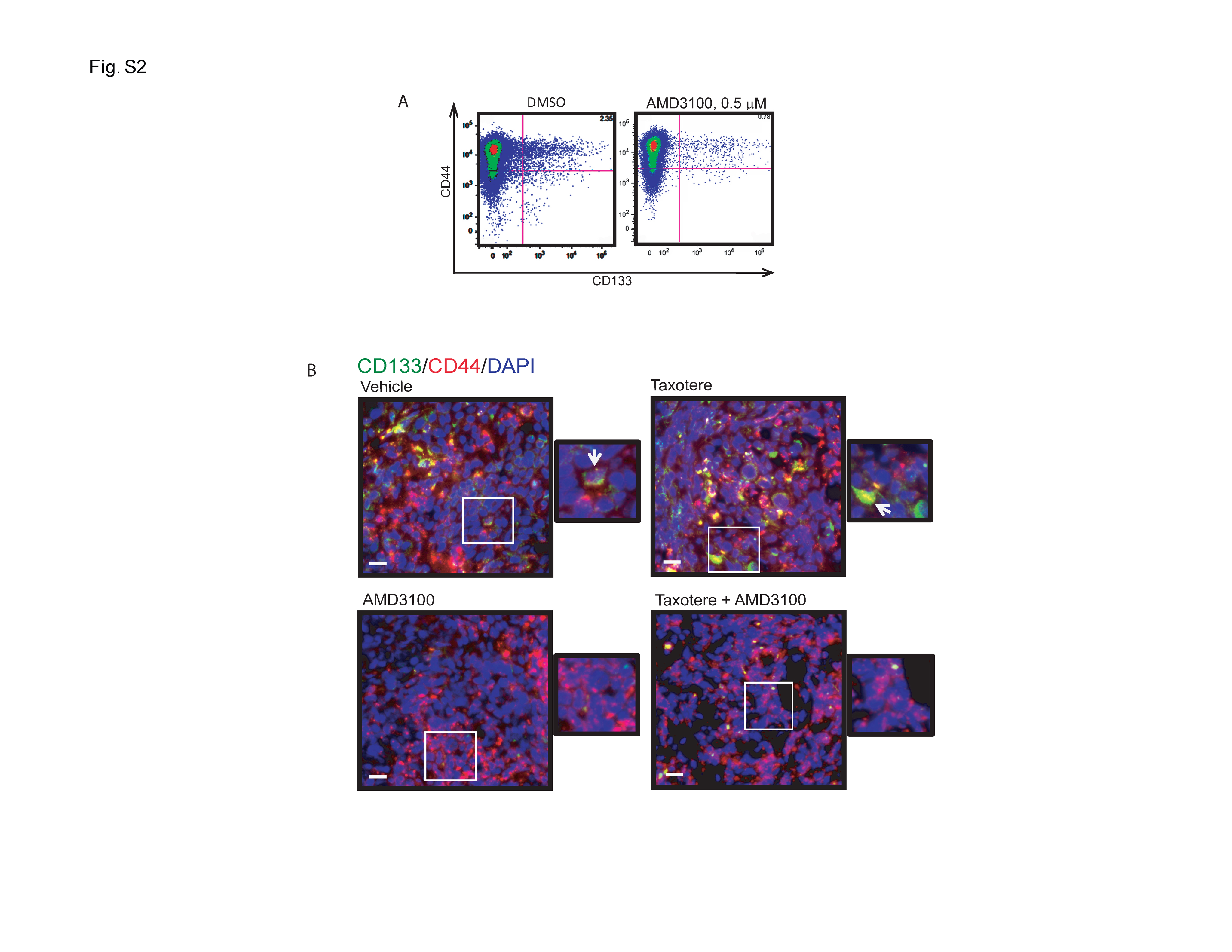

Supplement: Figure S2 — Targeting the tumor initiating population within DU145 and PC3 carcinoma cell lines by CXCR4 antagonist. (A) Treatment with CXCR4 antagonist AMD3100 decreases the CD133+/CD44+ population. PC3 cells were grown in serum-free, EBM medium with supplements and treated with 0.5 µM AMD3100. On the 5th day the cells were subjected to flow cytometry analysis. (B) CD133 and CD44 immunostaining on frozen sections of xenograft tumors treated with combinatorial or mono-therapy revealed selective inhibition of the CD133+/CD44+ population by the CXCR4 antagonist AMD3100. (TIF) [file pone.0031226.s002.tif]

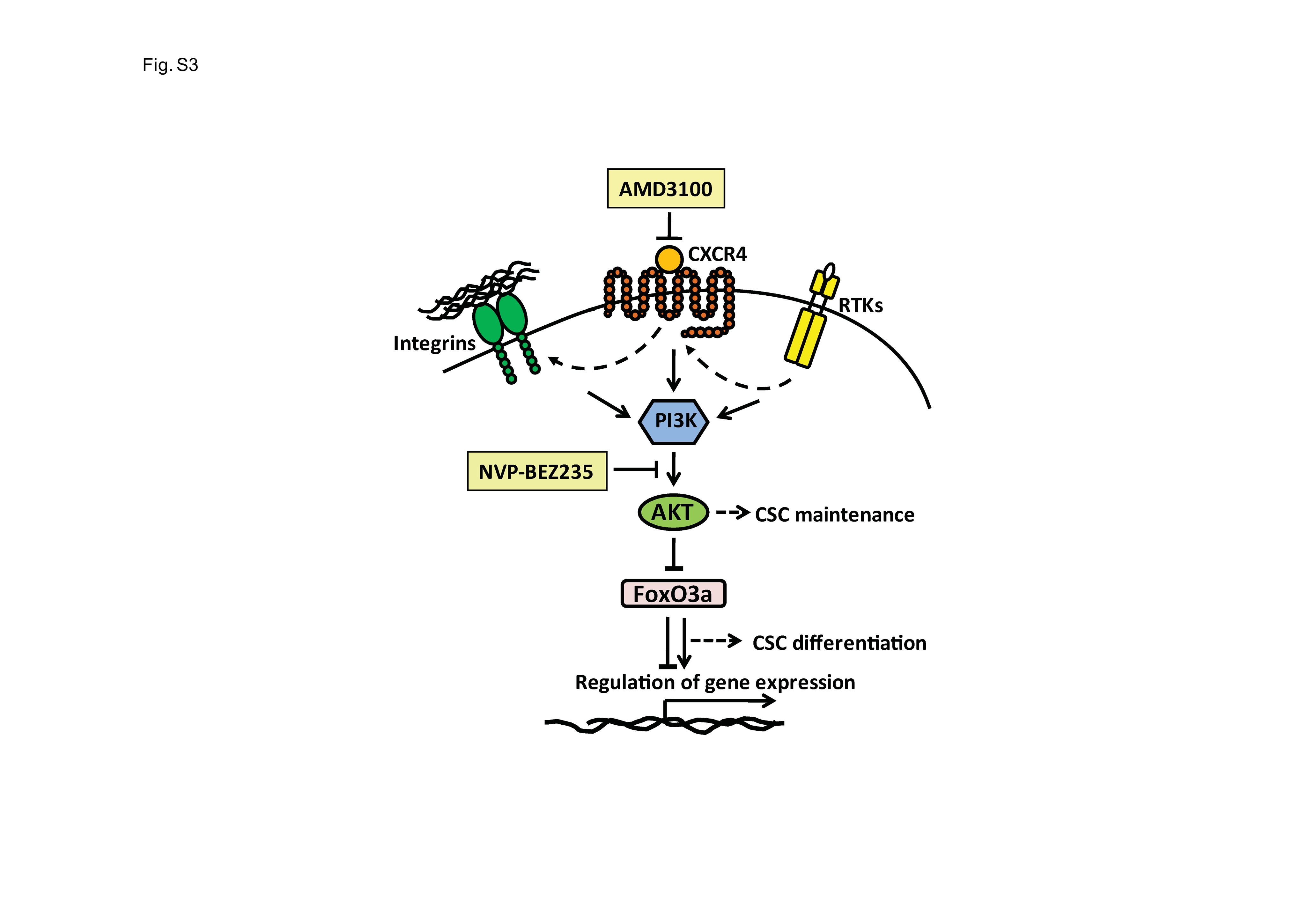

Supplement: Figure S3 — Mechanism of CXCR4/CXCL12 dependent maintenance of prostate cancer progenitors. CXCL4/CXCR12 – induced transactivation of receptor tyrosine kinases (EGFR, HER2, IGF-1R, FGFR, etc.) contributes to enhanced invasive signals and metastatic growth. The CXCL12-induced adhesion of prostate cancer progenitors to the extracellular matrix is mediated by integrins. PI3K pathway is one of the key mechanisms mediating the function of CXCR4 in prostate tumor initiating population. Targeting CXCR4 signaling with small molecule inhibitors may be beneficial in eliminating prostate cancer stem-like cells. (TIF) [file pone.0031226.s003.tif]

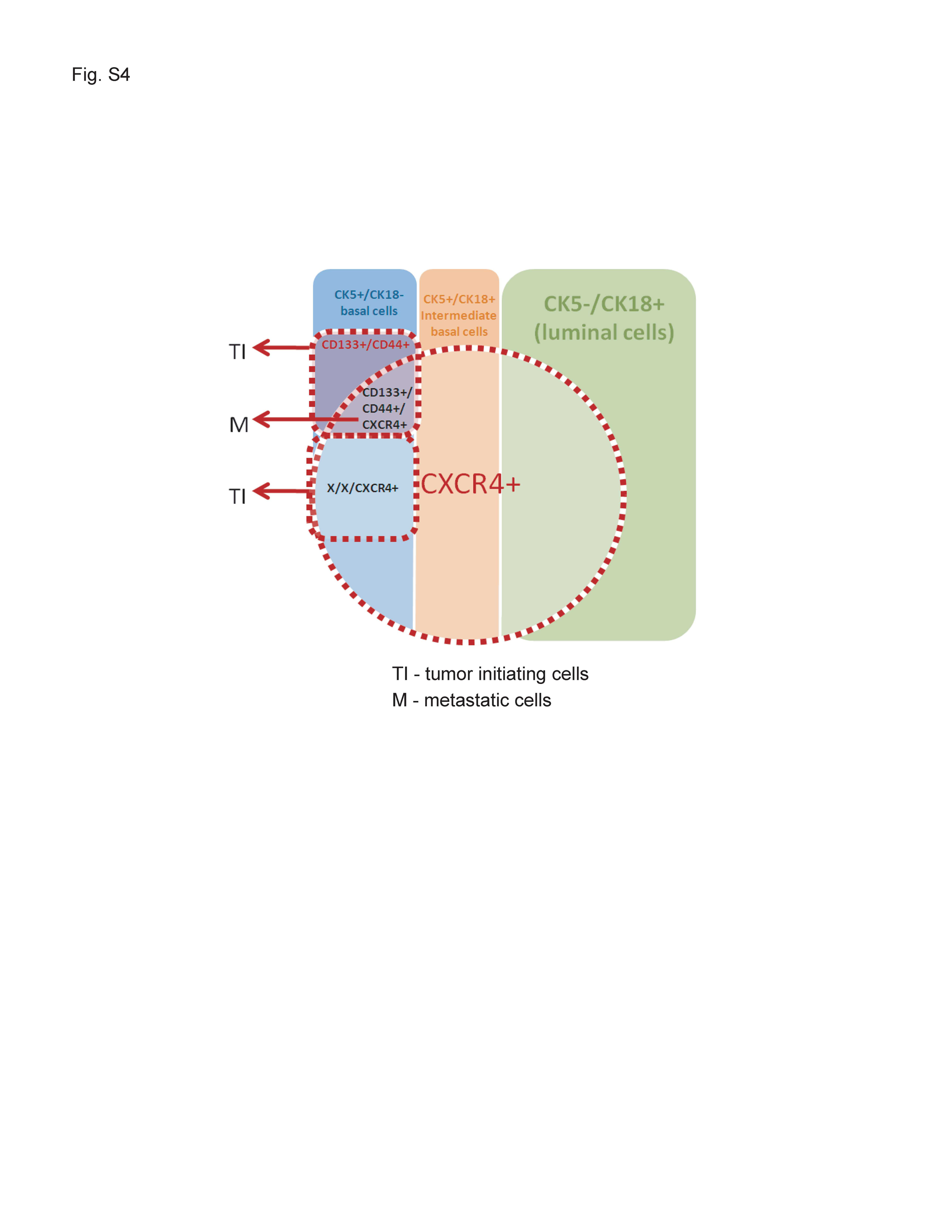

Supplement: Figure S4 — Model of prostate cancer cell heterogeneity. CXCR4+ cells represent a highly tumorigenic subset of cancer progenitors that could also have migratory properties. (TIF) [file pone.0031226.s004.tif]

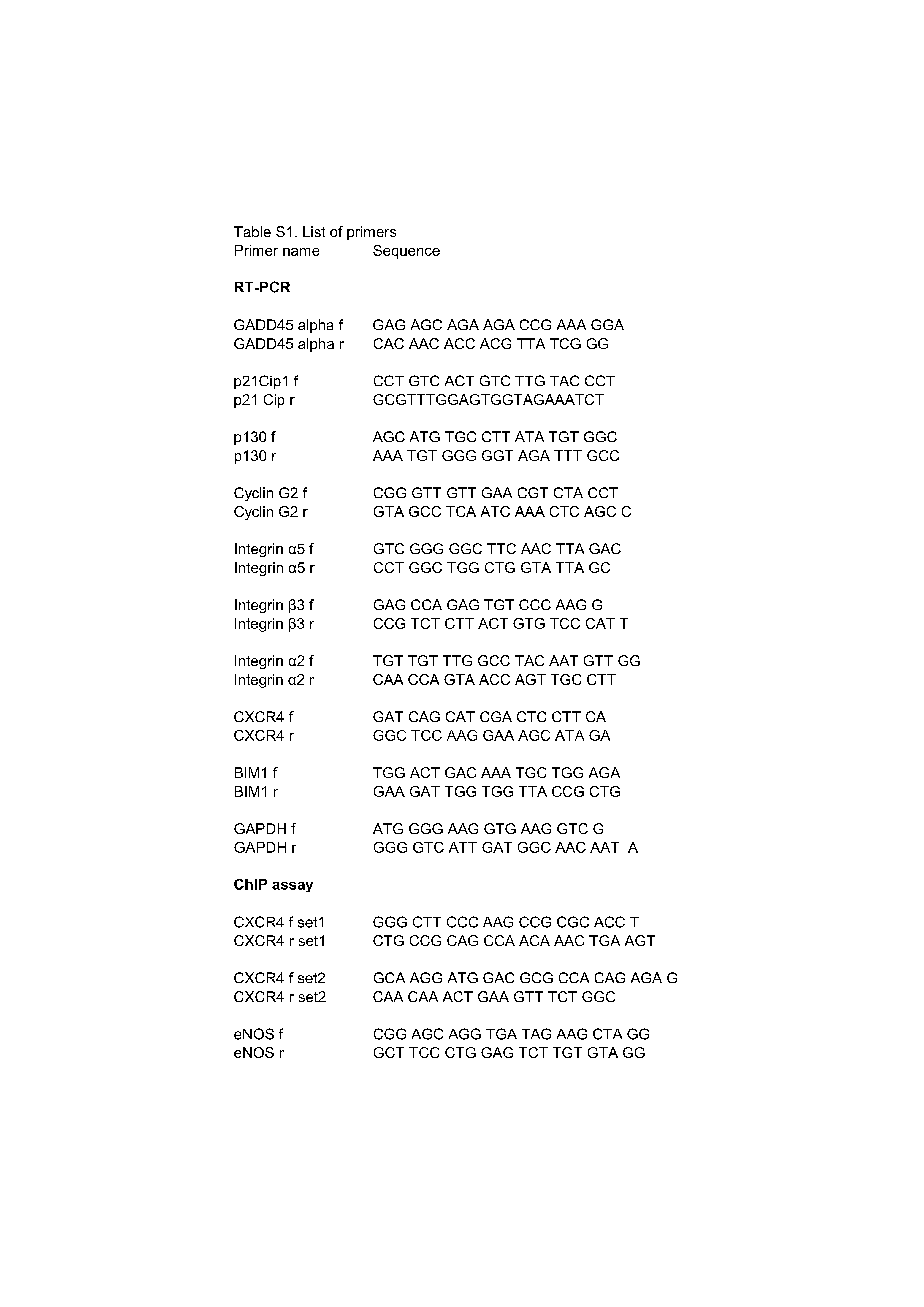

Supplement: Table S1 — List of primers used for RT-PCR and ChIP assay. (DOC) [file pone.0031226.s005.doc]
